# Alopecia areata following semaglutide treatment for weight loss: A case report

**DOI:** 10.1016/j.jdcr.2025.06.012

**Published:** 2025-06-19

**Authors:** Wasan S. Alzahrani, Sadeem A. Bahkali, Renad F. Alharthy, Ahmad S. Alsabban

**Affiliations:** a College of Medicine, King Saud Bin Abdulaziz University for Health Sciences, Jeddah, Saudi Arabia; b King Abdullah International Medical Research Center, Jeddah, Saudi Arabia; c Department of Family Medicine, Ministry of the National Gaurd-Health Affairs, Jeddah, Saudi Arabia

**Keywords:** adverse event, alopecia Areata, autoimmune disease, hair loss, semaglutide, weight loss

## Introduction

Semaglutide is a glucagon-like peptide-1 (GLP-1) receptor agonist initially used to manage type 2 diabetes.^1^^,^^2^ In recent years, semaglutide has gained significant attention for its effectiveness in facilitating weight loss.^1^^,^^2^ It mainly acts on the pancreas to stimulate insulin secretion, inhibit glucagon release, slow gastric emptying, and decrease appetite.^1^^,^^3^ However, alongside its benefits, it has been associated with some adverse effects. Specifically, one unknown adverse event is alopecia areata, which is a reversible loss of hair that is characterized by intact follicles.^4^ It significantly impacts patients’ lives not only in their physical self-esteem but also in their psychosocial health.^4^ This case report aims to describe the occurrence of alopecia areata in a patient undergoing treatment with semaglutide.

## Case presentation

A 23-year-old female presented with a sudden onset of a well-demarcated round patch of hair loss on her scalp, which she noticed approximately 2 months ago. She is a nonsmoker, medically healthy except for well-controlled asthma, for which she uses Symbicort (budesonide/formoterol) 160 mcg for maintenance and salbutamol 100 mcg as needed, with no exacerbations in the past 3 years. She is active and has no significant psychosocial stressors in her life. In particular, past medical history is negative for skin or hair disorders, thyroid disease, and malignancy. The patient’s family history was noncontributory except for an uncle with a diagnosis of Graves’ disease and a brother with asthma. The patient’s previous body mass index was approximately 30.20 kg/m^2^, which classified her as obese. Prior to her hair presentation, the patient had been using semaglutide for weight loss over the past 4 months and lost 10 kg. Her iron-deficiency anemia was corrected before starting semaglutide. Initially, she started on 0.25 mg once weekly for the first 2 months, followed by 0.5 mg once weekly during the third and fourth months. Notably, the first single well-demarcated round patch of hair loss appeared during the third month of semaglutide treatment, after the dose was increased to 0.5 mg. The patient first noticed it on the crown area (approximately 1.1 cm in size), which developed gradually without associated symptoms like pain, redness, or itching. Following the next month, the round patch of hair loss has increased in size, and other similar patches have developed, prompting the patient to seek medical attention.

Upon physical examination at the dermatology clinic, there was slight redness, no scaling, or any noticeable changes in the scalp; however, the patient was found to have multiple nonscarring patches of well-demarcated round areas of hair loss. These included the original crown patch, currently increased to 2 × 3 cm, along with new patches in the occipital area (3 × 3 cm), left temporal region (1 × 1 cm), and the frontal area near the hairline (1 × 1 cm). Her remaining physical examination was unremarkable, with no signs of nail pitting or hair loss in other hair-bearing areas. The dermatoscopy and hair pull tests were positive with exclamation point shafts and some hair falling, respectively. After the clinical diagnosis of alopecia areata, the patient was advised to discontinue semaglutide immediately after completing 4 months of its course. Alopecia areata management was then initiated, consisting of 1.5 cc 5 mg intralesional corticosteroid injections (triamcinolone acetonide) administered every 6 weeks into the affected areas. Additionally, the patient was prescribed ketoconazole 2% shampoo to be used 3 times a week to help reduce inflammation and minoxidil 5% topical solution applied daily to the areas of hair loss.

**Fig 1:**
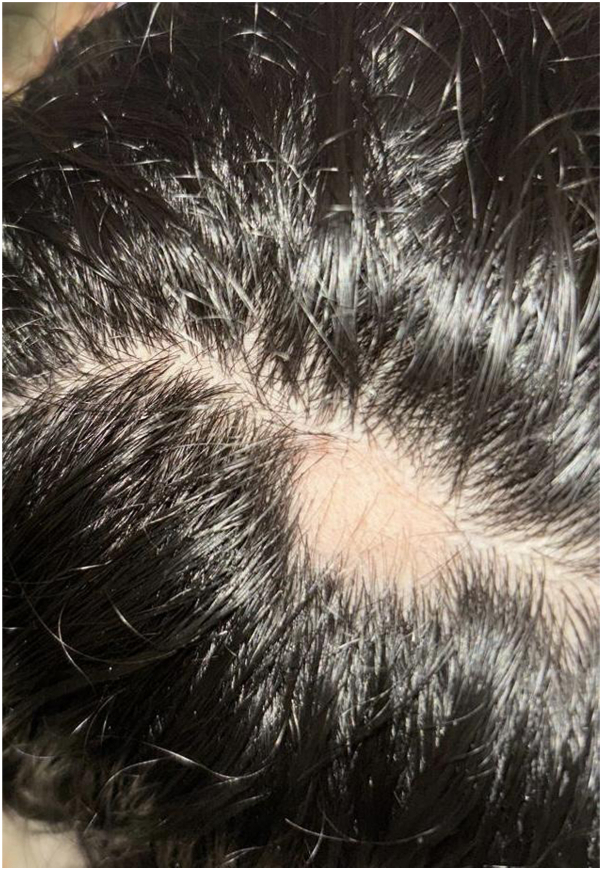
Crown patch measuring 4 × 4 cm following semaglutide treatment.

**Fig 2:**
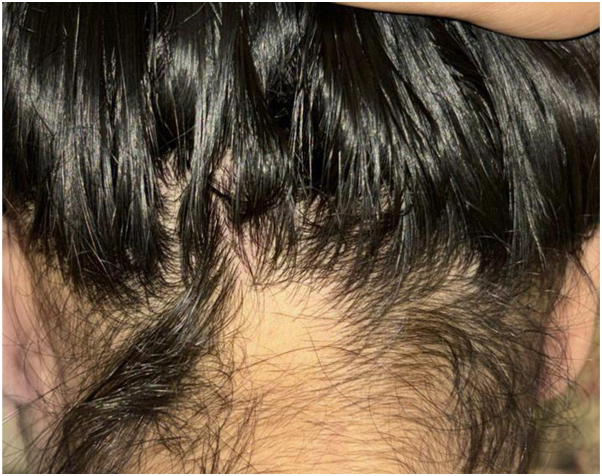
Occipital patch measuring 3 × 4 cm following semaglutide treatment.

**Fig 3:**
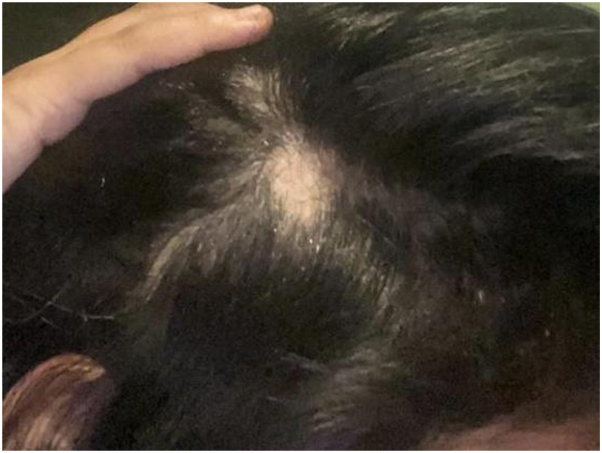
Left temporal patch measuring 2 × 1 cm following semaglutide treatment.

**Fig 4:**
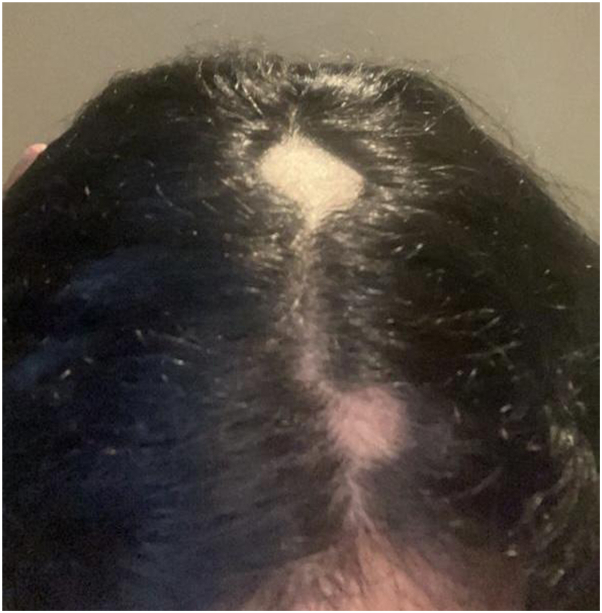
Frontal patch measuring 2 × 2 cm following semaglutide treatment.

Over the course of the first 12 weeks, following the first and second injection sessions, no significant improvement was visible. The hair loss skin patches continued to increase in size, with the crown patch measuring 4 × 4 cm (Fig 1), the occipital patch measuring 3 × 4 cm (Fig 2), the temporal patch at 2 × 1 cm (Fig 3), and the frontal patch at 2 × 2 cm (Fig 4). However, the patient showed significant improvement and treatment response between the third and fourth intralesional corticosteroid injection sessions. The occipital and temporal patches showed complete resolution, while a notable regrowth was seen in the frontal and crown patches. By the time of the fifth injection session, there was further improvement in the crown patch and near-complete regrowth of hair in all affected patches.

## Discussion

Semaglutide received Food and Drug Administration approval in 2017 for the management of type 2 diabetes and was later approved in 2021 for weight management.^1^ Recently, semaglutide has become the first-line option for patients opting for nonsurgical weight loss management. GLP-1 is a natural hormone predominantly produced in the small intestine, with minor synthesis occurring in the pancreas and the brain.^3^ It plays a key role in controlling blood sugar by promoting insulin release and reducing glucagon secretion when glucose levels are increased.^1^^,^^2^ Drugs like semaglutide are designed to mimic the natural GLP-1 hormone, with modifications that allow it to remain active in the body for longer.

Various causes could disrupt the hair growth cycle, such as genetic predisposition, autoimmune disorders, hormonal imbalances, stress, and medications.^4^ Driven by some recent cases, there is a significant link between alopecia and the use of semaglutide.^5^^,^^6^ This association is not yet fully understood; however, it may be attributed to substantial nutritional deficiencies resulting from reduced food intake.^4^ Similarly, rapid weight loss can disrupt normal nutritional balance, potentially exacerbating hair loss.^4^ A recent study indicated that individuals undergoing bariatric surgery may experience similar hair loss patterns.^7^ This suggests that the mechanism behind hair loss could be related to the rapid changes in the body that accompany both semaglutide treatment and bariatric procedures.^8^ Furthermore, alopecia has been identified as a potential adverse effect of semaglutide, as noted in a few recent studies that discuss dermatologic adverse events associated with the medication.^9^^,^^10^ Given the novelty of this topic, the existing literature is limited; however, a new study has shown an analysis of the Food and Drug Administration Adverse Event Reporting System database which reveals that between 2022 and 2023, there were 199 reports of alopecia linked to semaglutide and other GLP-1 and GIP/GLP-1 agonists.^9^ There is a lack of direct evidence, highlighting the need for further research to deepen our understanding of the association between semaglutide and alopecia areata.

## Conclusion

This case emphasizes the importance of monitoring for signs of alopecia areata in patients undergoing rapid weight loss with semaglutide. Further research is needed to clarify the relationship between semaglutide and alopecia areata, but clinicians should be aware of this potential adverse effect when managing patients on this treatment. Finally, it should be acknowledged that the onset of alopecia areata could be coincidental given the idiopathic nature of the disease.

## Conflicts of interest

None disclosed.
